# Frequency of Medication Non-compliance in Hypertensive Patients Presenting with Stroke: A Case-control Study

**DOI:** 10.7759/cureus.4605

**Published:** 2019-05-07

**Authors:** Vikash Kumar, Barkha Kumari, Eiman Rahat, Sundus Fareed

**Affiliations:** 1 Internal Medicine, Jinnah Sindh Medical University, Karachi, PAK; 2 Internal Medicine, Ziauddin University, Karachi, PAK; 3 Internal Medicine, Civil Hospital, Karachi, PAK

**Keywords:** morisky medication adherence scale, hypertension, medication compliance, drug adherence, hypertension related stroke, complications of hypertension

## Abstract

Introduction

Medication compliance (MC) is essential for optimum control and delaying disease progression and complications in chronic illnesses. Patients with hypertension have been repeatedly studied for their pattern of MC in the literature. However, whether or not lack of MC is an issue grave enough to cause medical complications of hypertension is still not clear. The aim of this study was to evaluate if the lack of MC is related to hypertension-related stroke.

Methods

In this case-control, observational study, 100 hypertensive patients admitted with hypertensive stroke were included. These cases were compared with 200 hypertensive patients without any major hypertensive complication recruited from outpatient clinics. Medication compliance was calculated using the Morisky Medication Adherence Scale (MMAS). Data was entered and analysed using SPSS v. 22.0.

Results

High compliance patients were more in the control group than the cases (34.5% vs. 27%), similar was with medium (41.5% vs. 30%). and low compliance patients (43% vs. 24%; *p *= 0.003). In both high compliant and moderate-to-low compliant group, mean systolic and diastolic blood pressure was higher among the cases (*p* <0.05). Among high compliant patients, cases were taking more pills per day than the controls (*p *= 0.032). Among moderate-to-low compliant patients, 80% perceived themselves to be highly compliant and only 20% perceived to be low complaint in the cases, as compared to 60% controls perceiving themselves compliant and 40% as low complaint (*p *= 0.001).

Conclusion

The incidence of low medication adherence is significantly higher in patients with major hypertensive complications such as stroke as compared to hypertensive patients without any major complication.

## Introduction

Chronic non-communicable diseases are alone responsible for 71% of global mortality, according to the World Health Organization (WHO). The most common metabolic change predisposing to non-communicable diseases is elevated blood pressure which alone is attributed to 19% of global deaths related to non-communicable diseases [1]. “Elevated blood pressure” was defined as systolic pressure 120-129 mm Hg even when diastolic pressure less than 80 mm Hg by the American College of Cardiology/American Heart Association (ACC/AHA) High Blood Pressure Guidelines, in 2017. For Stage I and II hypertension, systolic pressure is taken as 130-139 mm Hg and ≥140 mm Hg respectively, and diastolic is taken as 80-89 mm Hg and ≥90 mm Hg, respectively [2]. According to WHO, one in every four adults in Pakistan is hypertensive [3]. Although no nationwide study has been conducted to evaluate the prevalence of hypertension (HTN) in Pakistan, various studies show that it ranges from 26% to 39% in adults [4-6].

Since essential hypertension is multi-factorial, its treatment approach is also multi-faceted. Management of HTN involves, adopting a healthy and physically active lifestyle along with add-on drug therapy [7]. As with all other chronic progressive diseases, compliance to a healthy lifestyle and medication regimen are essential to slow down disease progression and delay the onset of complications [8]. Adherence is not about timely consumption of pills but it reflects an overall healthy lifestyle. In an Ethiopian study, only 23% of the hypertensive patients were compliant to the lifestyle manifestations advised by their physicians [9]. In a large-scale, longitudinal cohort of hypertensive patients without any cardiac risk factor, there was a 37% reduced risk of cardiovascular outcome in patients who were compliant to their anti-hypertensive therapy [10].

In another retrospective cohort, as the medication possession ratio reduced in hypertensive patients, their risk of cerebrovascular accidents increased from 1.36-2 times [11]. Compliance to therapy is critical for the prevention of complications in patients with chronic progressive illnesses. Medication non-compliance is a manageable issue. If medication non-compliance actually has a relationship with emergency events in chronic illnesses, addressing non-compliance can achieve greater disease control and reduce the incidence of acute events. We aimed to evaluate if there is any relationship between medication compliance (MC) and cerebrovascular complications among hypertensive patients by comparing MC in patients who have developed a cerebrovascular complication with those who have not. 

## Materials and methods

Study design, settings, and sample

It was an observational case-control study conducted in a tertiary care centre in Karachi. Institutional review board approval was attained and written informed consent was attained from all participants. The sample size was calculated using OpenEpi for control to case ratio 2:1 and odds ratio 2 [12]. The sample size for cases was calculated to be 100 and for controls 199.

“Controls” were defined as patients with known hypertension as defined in ACC/AHA guidelines [3]. They were recruited from the outpatient department (OPD) of the hospital. Patients who have had a one or more previous episodes of hypertensive strokes were excluded.

“Cases” were defined as known hypertension patients admitted in the internal medicine department with the clinical and neuro-radiological diagnosis of stroke by the attending physician. Only patients with ischemic or haemorrhagic as a result of raised blood pressure were included. Stroke patients with causes other than raised blood pressure were excluded.

Study instrument

A structured performa was created. It included two sections. The first section comprised of patient age, gender, duration of diagnosis of hypertension, blood pressure in mmHg and body mass index (BMI) in kg/m^2^ (within the OPD for controls and at the time of admission for cases), total number of daily pills taken, self-rated health status (5: excellent, 4: very good, 3: good, 2: fair, 1: poor), and self-rated MC (3: high, 2: moderate, 1: low).

The second section of the performa comprised Modified Morisky Adherence Scale (MMAS). It is an eight-item validated instrument used to assess the extent of drug adherence. With a maximum score of 8, it classifies patients into highly compliant (score: 8), moderately compliant (score: 6 - <8), and low compliant (score: <6) [13].

Data analysis

Data was entered and analysed using Statistical Package for Social Sciences (SPSS) version 22.0 (NY, USA). Categorical data was analysed in terms of frequency and percentages. Continuous data were analysed in terms of mean and standard deviation (SD). For the sake of data interpretation, patients who scored <8 on MMAS were combined into one group - moderate to low compliant. Similarly, scores 1 and 2 on self-rated MC were combined into one group - moderate to low adherent (score: 1-2) and score 4 and 3 on self-rated health status were combined into one group - very good to good (score: 3-4). The extent of MC as recorded on MMAS was compared with patient factors. Chi-square was applied to find the statistical correlation between the extent of medication compliance and related factors. *P*-value of ≤0.05 was considered significant.

## Results

In the control group, there were 92 (46%) men and 108 (54%) women. Their mean age was 54.34 ± 6.07 years and their mean duration of hypertension was 4.13 ± 2.03 years. The mean BMI was 29.94 ± 2.44 kg/m2. Their mean systolic blood pressure was 129.46 ± 7.25 and diastolic was 110.33 ± 4.56. In the cases group, there were 77 (77%) men and 23 (23%) women. Their mean age was 59.47 ± 3.55 years and their mean duration of hypertension was 4.43 ± 1.85 years. The mean BMI was 29.08 ± 3.72 kg/m2. Their mean systolic blood pressure was 179.50 ± 8.79 and diastolic was 123.53 ± 6.92.

The MMAS scores of cases and controls are shown in Table 1. High MC patients were more in the control group than the cases (34.5% vs. 27%), similar was with medium MC patients (41.5% vs. 30%). Low MC patients were common in the cases group (43% vs. 24%). The differences were statistically significant (*p *= 0.003; Table 1).

**Table 1:** Extent of medication compliance of cases and controls as assessed by MMAS MMAS, Modified Morisky Adherence Scale

Medication compliance on MMAS	Control group (n = 200) (%)	Cases group (n = 100) (%)	P value	Total patients (n = 300)
High	69 (34.5%)	27 (27.0%)	0.003	96 (32.0%)
Medium	83 (41.5%)	30 (30.0%)		113 (37.7%)
Low	48 (24.0%)	43 (43.0%)		91 (30.3%)

In the control group, there were 70 (35%) patients consuming 1-3 pills daily, 41 (20.5%) patients consuming 4-6 pills daily, and 89 (44.5%) were consuming >6 pills per day. There were 24 (12%) patients who self-rated their health as “excellent,” 62 (31%) rated their health as “very good-good,” 55 (27.5%) rated their health as “fair,” and 59 (29.5%) rated their health as “poor.” There were 136 (68%) patients who self-rated their drug adherence as “high” and 64 (32%) self-reported their drug adherence as “moderate-to-low”.

In the cases group, there were 18 (18%) patients consuming 1-3 pills daily, 33 (33%) patients consuming 4-6 pills daily, and 49 (49%) were consuming >6 pills per day. There were 8 (8%) patients who self-rated their health as “excellent,” 27 (27%) rated their health as “very good-good,” 31 (31%) rated their health as “fair,” and 34 (34%) rated their health as “poor.” There were 81 (81%) patients who self-rated their MC as “high” and 19 (19%) self-reported their MC as “moderate-to-low”.

When the cases and controls were divided according to their extent of MC, it was seen that 27 (27%) cases were high compliant and 73 (73%) were moderate-to-low compliant. In the control group, 69 (34.5%) were high compliant and 131 (65.6%) were moderate-to-low compliant. The factors that can affect the medication compliance are compared to the actual compliance of cases and controls in Table 2. In the cases group of high compliant participants, there were 21 (77.7%) men and 6 (22.2%) women as compared to 33 (47.8%) men and 36 (52.2%) women in the controls group (*p *= 0.007). There was no statistical significance of age and duration of hypertension in both high compliant and moderate-to-low compliant. The mean of both systolic and diastolic blood pressure was statistically significant for both high compliant and moderate-to-low compliant groups. BMI was only statistically significant for men and women in moderate-to-low compliant group. The total number of daily pills was only statistically significant for moderate-to-low compliant group. Self-rated health status did not show any statistically significant relationship with MC. Self-reported MC was statistically significant for moderate-to-low compliant group (Table 2).

**Table 2:** Factors affecting medication compliance compared to the actual compliance of the patients as assessed by Modified Morisky Adherence Scale SD, standard deviation; OPD, outpatient department

	High medication compliance (n = 96; 32%)			Moderate to low medication compliance (n = 204; 68%)		
	Cases (n = 27)	Controls (n = 69)	p value	Cases (n = 73)	Controls (n = 131)	p value
Gender						
Male	21 (77.7%)	33 (47.8%)	0.007	56 (76.7%)	59 (45.0%)	0.000
Female	6 (22.2%)	36 (52.2%)		17 (23.3%)	72 (55.0%)	
Age						
40-55 years	11 (40.7%)	27 (39.1%)	0.88	40 (54.8%)	82 (62.5%)	0.275
>56 years	16 (59.3%)	42 (60.8%)		33 (45.2%)	49 (37.4%)	
Duration of hypertension						
0-3 years	10 (37.0%)	35 (50.7%)	0.189	5 (6.8%)	18 (13.7%)	0.292
3-6 years	10 (37.0%)	26 (37.7%)		33 (45.2%)	59 (45.0%)	
> 6 years	7 (26.0%)	8 (11.6%)		35 (47.9%)	54 (41.2%)	
Mean ± SD blood pressure at admission / in OPD (mmHg)						
Systolic	159.85 ± 11.79	138.24 ± 6.07	<0.000	184.33 ± 9.24	130.77 ± 9.02	<0.000
Diastolic	119.25 ± 7.58	115.22 ± 7.53	0.020	124.82 ± 10.44	109.23 ± 12.83	<0.000
Mean ± SD body mass index at admission / in OPD (kg/m^2^)						
Males	34.58 ± 2.88	34.20 ± 3.04	0.38	33.52 ± 2.04	32.15 ± 2.11	0.004
Female	34.11 ± 2.77	33.89 ± 2.53	0.56	34.54 ± 1.55	32.22 ± 2.74	0.000
Number of pills consumed per day						
1–3	3 (11.1%)	24 (34.7%)	0.032	15 (20.5%)	46 (35.1%)	0.070
4–6	9 (33.3%)	11 (15.9%)		24 (32.8%)	30 (22.9%)	
> 6	15 (55.5%)	34 (49.3%)		34 (46.5%)	55 (42.0%)	
Self rated heath status						
Excellent	3 (11.1%)	7 (10.1%)	0.422	5 (6.8%)	17 (12.9%)	0.120
Very good – good	11 (40.7%)	19 (27.5%)		16 (22.0%)	43 (32.8%)	
Fair	3 (11.1%)	17 (24.6%)		28 (38.4%)	38 (29.0%)	
Poor	10 (37.0%)	26 (37.8%)		24 (32.8%)	33 (25.2%)	
Self-rated drug adherence						
High	22 (81.4%)	58 (84.1%)	0.760	59 (80.8%)	78 (59.5%)	0.001
Moderate – low	5 (18.5%)	11 (15.9%)		14 (19.2%)	53 (40.5%)	

## Discussion

In this comprehensive study, hypertensive cases that developed cerebrovascular incident showed significantly reduced medication compliance as compared to the control group. Even in their high compliance group, the cases had higher mean blood pressure and BMI than the controls. Along with this, the cases were consuming a higher number of total daily pills. The self-rated health status was comparable in both cases and controls of the high compliant and moderate-to-low compliant groups. In the moderate-to-low compliant group, more cases self-reported their MC to be high as compared to the controls; the difference was statistically significant.

This study is substantial in adding to the growing literature highlighting a crucial relationship between cerebrovascular complications in hypertensive patients who were not compliant to their medications. However, it was a small-sample, single-center study, which took place at one point in time; hence, any casual relationship cannot be deduced. Patients were included in the study when they already have developed the adverse outcome (stroke), hence, this retrospective method is subjected to recall bias. This study assessed medication compliance through a standard, validated research instrument which is a moderately accurate method. More accurate methods of measuring medication compliance including pill counting, prescription record review, electronic monitoring, and directly observed therapy must be applied for more accurate results [14].

In a Chinese study with HTN patients discharged after stroke, 35% were found to be compliant to their doctor’s prescription. Poor MC in their patients was related to female gender, unemployed participants, participants with stage III HTN, participants with poor HTN knowledge, and participants who did not monitor their blood pressure regularly [15]. Comparatively, in this study, the factors associated with moderate-to-low medication compliance in stroke patients were male gender, uncontrolled blood pressure, high BMI, and self-rated high MC.

Results on medication compliance from the REasons for Geographic And Racial Disparities in Stroke (REGARDS) trial also showed low medication compliance in female patients with limited income resources. Patients with low medication compliance in the REGARDS trial had 1.08 times greater risk of stroke [16]. Another study with a similar case-control sample as ours showed that in HTN patients who developed stroke the highest odds ratio (OR) upon logistic regression was seen with “medication not taking as prescribed” (OR = 6.07) [17]. Hypertensive patients non-compliant to treatment showed a higher frequency of cerebrovascular accidents as compared to the ones who were compliant (25% vs. 13%; OR: 3.04; *p *<0.001) [18]. In a study with hypertensive stroke patients, 17% were highly compliant of Morisky scale, 6% were moderately compliant and 77% had low medication compliance. Their main reasons were lack of knowledge and high cost. Only 3.6% of patients reported pill burden as a reason to low MC in their study [19].

This study has shown a crucial relationship with reduced medication compliance and the incidence of cerebrovascular accidents in hypertensive patients. Since this study was conducted at one point in time, casual relationship cannot be established. However, we recommend longitudinal studies with larger sample size to study this predictor in depth. Medication non-compliance can be taken as a reversible risk factor for acute events in chronic illnesses. Physicians should maintain a healthy bond with their patients, press the need of medication compliance to their patients, and most importantly aware them about the progression of their disease and consequences of non-compliance. Awareness lets the patients make an informed choice about their attitude towards the disease. 

## Conclusions

Hypertension is a chronic illness that requires a multifactorial approach to management. A combination of healthy lifestyle and adequate pharmacologic interventions are essential to keep blood pressures in optimum range and prevent any major organ complications. Patients with major hypertensive complications including stroke are associated with low medication adherence in this study. These patients reported higher BMI and daily pill count and perceived their medication compliance to be higher which actually was not.
